# Differences in Neural Recovery From Acute Stress Between Cortisol Responders and Non-responders

**DOI:** 10.3389/fpsyt.2018.00631

**Published:** 2018-11-26

**Authors:** Annika Dimitrov, Katharina Demin, Phöbe Fehlner, Henrik Walter, Susanne Erk, Ilya M. Veer

**Affiliations:** ^1^Research Division of Mind and Brain, Department of Psychiatry and Psychotherapy CCM, Charité–Universitätsmedizin Berlin, Corporate Member of Freie Universität Berlin, Humboldt-Universität zu Berlin, and Berlin Institute of Health, Berlin, Germany; ^2^Mood Disorders Research Group, Department of Psychiatry and Psychotherapy CCM, Charité–Universitätsmedizin Berlin, Corporate Member of Freie Universität Berlin, Humboldt-Universität zu Berlin, and Berlin Institute of Health, Berlin, Germany; ^3^Department of Epileptology, Epilepsy-Center Berlin-Brandenburg, Evangelisches Krankenhaus Königin Elisabeth Herzberge, Berlin, Germany; ^4^Division of Systems Neuroscience in Psychiatry, Department of Psychiatry and Psychotherapy, Central Institute of Mental Health, Medical Faculty Mannheim, University of Heidelberg, Mannheim, Germany

**Keywords:** stress recovery, fMRI, resting state, functional connectivity, amygdala, cortisol

## Abstract

Adaptive recovery from a stressor fosters resilience. So far, however, few studies have examined brain functional connectivity in the aftermath of stress, with inconsistent results reported. Focusing on the immediate recovery from psychosocial stress, the current study compared amygdala resting-state functional connectivity (RSFC) before and immediately after psychosocial stress between cortisol responders and non-responders. Differences between groups were expected for amygdala RSFC with regions involved in down-regulation of the physiological stress response, emotion regulation, and memory consolidation. Eighty-six healthy participants (36 males/50 females) underwent a social stress paradigm inside the MRI scanner. Before and immediately after stress, resting-state (RS) fMRI scans were acquired to determine amygdala RSFC. Next, changes in connectivity from pre- to post-stress were compared between cortisol responders and non-responders. Responders demonstrated a cortisol increase, higher negative affect, and decreased heart rate variability (HRV) in response to stress compared to non-responders. A significant Sex-by-Responder-by-Time interaction was found between the bilateral amygdala and posterior cingulate cortex (PCC) and precuneus (*p* < 0.05, corrected). As males were also more likely to show a cortisol increase to the stress task than females, follow-up analyses were conducted for both sexes separately. Whereas no difference was observed between female responders and non-responders, male non-responders showed an increase in FC after stress between the bilateral amygdala and the PCC and precuneus (*p* < 0.05, corrected). The increased coupling of the amygdala with the PCC/precuneus, a core component of the default mode network (DMN), might indicate an increased engagement of the amygdala within the DMN directly after stress in non-responders. Although this study was carried out in healthy participants, and the results likely reflect normal variations in the neural response to stress, understanding the mechanisms that underlie these variations could prove beneficial in revealing neural markers that promote resilience to stress-related disorders.

## Introduction

While we plan, set goals, and build expectations concerning our present and future, we are confronted with numerous situations that challenge our resources and our prospect of life. Both predictable and unpredictable events require a continuous adaptation to regain balance on a physiological and psychological level. McEwen and Wingfield (1) described this adaptive process as maintaining stability in life-essential systems (“homeostasis”) through change (“allostasis”). The allostatic state is reflected by the adjustment or maintenance of physiological and behavioral systems in order to adapt to challenging or stressful situations. An imbalance in these physiological systems over a prolonged period of time may result in allostatic overload and in the long run in stress-related psychopathology, such as major depressive disorder or posttraumatic stress disorder (2), depending on individual experiences, genetic predispositions, and social factors. Studying the mechanisms supporting adaptive recovery from stress is thus of importance, as this may ultimately improve interventions aimed to maintain resilience after adversity.

Early work has mainly focused on the physiological stress response, which comprises an immediate and a delayed response. The immediate reaction is elicited by the activation of the sympatho-adrenomedullary pathway of the autonomic nervous system. It expresses itself in rapid physiological effects, caused by the release of epinephrine and norepinephrine from the adrenal medulla. The resulting autonomic alternations are typically known as the fight-or-flight response (3), and are directed toward preparing the organism to deal with a threatening or stressful situation. In general, the autonomic response is short-lived, as the parasympathetic nervous system—the antagonist of the sympathetic nervous system—exerts regulatory control after a short while (4). The neuroendocrine response entails a delayed secretion of glucocorticoids. Through the activation of the hypothalamus-pituitary-adrenal (HPA) axis, a sequence of different physiological processes leads to the release of glucocorticoids (GCs). The most important glucocorticoid in humans is the stress hormone cortisol (4). In general, GCs play a very heterogeneous role in stress, as they can serve permissive, stimulative, suppressive, and preparative functions (5). With respect to recovery from stress, two functions of GCs are especially of interest: First, GCs regulate their own secretion by acting back on the HPA axis in a negative feedback loop, thereby inhibiting further secretion of adrenocorticotropic hormone (ACTH) (6). Second, GCs are crucial in processes of memory consolidation, facilitating learning of emotional information (7).

An important function of the stress response is to prepare the organism for future stressful experiences by promoting the memory consolidation of current stressful events (7). Encountering a stressful situation in the future enables the organism for an adaptive stress response, as it can revert to stored contextual information from previous and similar stressful experiences. A well-known phenomenon that reflects this adaptive function of the stress response is that emotionally significant events are indeed more likely to be remembered (7–9). Stress agents, such as norepinephrine and cortisol, are involved in the enhanced memory consolidation of emotional information, as they directly influence the activation of brain structures supporting memory (7). Moreover, there is evidence that the amygdala interacts with the hippocampus in mediating the effects of stress on the consolidation of contextual information (7, 10). As a second function of the stress response, negative emotions, which often follow stressful experiences, need to be adjusted as part of emotion regulation. Therefore, emotion regulation initiates a more regulatory role in the stress response to allow the return to the initial state of homeostasis. Specifically, the interactions between the amygdala and medial PFC (mPFC) are deemed essential for successful emotion regulation and may be mediated by cortisol, as these interactions seem to strengthen after hydrocortisone administration (11), and were related to endogenous cortisol fluctuations as well (12).

Over the past decade, neuroimaging methods have given us more insight into the underlying neural mechanisms involved in the stress response (13, 14), primarily focusing on the activity of the amygdala during the stress experience or immediately thereafter. During stress, a decrease in the activity of limbic structures was found, including the amygdala, mPFC, and hippocampus (15, 16). In contrast, the amygdala showed increased reactivity to emotionally negative stimuli in the aftermath of psychological stress (17, 18). However, to understand how brain regions interact with each other in initiating and regulating stress responses, we need to resort to measures of connectivity between remote brain regions rather than assessing activation in single areas. Resting-state (RS) fMRI might be the most intuitive paradigm to study connectivity changes in the aftermath of stress, as it assumes diffuse mind states and allows a rather “naturalistic” and undirected assessment of neural recovery mechanisms. So far, only five studies have examined the effects of psychological stress on resting-state functional connectivity (RSFC) of the brain in healthy volunteers.

Van Marle et al. (19) studied amygdala RSFC following a stress induction paradigm, in which participants had to watch aversive film clips. The comparison between the stress and control group revealed increased RSFC of the amygdala with the dorsal anterior cingulate cortex (dACC), the anterior insula (AI), and a dorso-rostral pontine region after stress. As the dACC and AI are both involved in mediating autonomic responses, the connectivity pattern obtained was interpreted to represent a vigilant state following the stress induction.

In the second study, Vaisvaser et al. (20) studied a more fine-grained trajectory of the stress response in the brain, using a serial subtraction arithmetic task. They compared RSFC of two different seed regions, the PCC and hippocampus, between three different time-points: before stress, immediately after stress, and 2 h after stress. Immediately after stress, the PCC increased its functional connectivity with the following regions: mPFC, thalamus, caudate nucleus, and inferior parietal lobule. This RSFC pattern was reversed when measured 2 h after stress. In contrast, an increased RSFC between the hippocampus and amygdala following stress persisted up to 2 h, pointing to a prolonged effect of stress on RSFC of the brain. Moreover, the authors found that non-responders specifically were characterized by a sustained increase in connectivity between these limbic regions.

Quaedflieg et al. (21) assessed RSFC before, immediately after, and 30 min after stress induction with the Maastricht Acute Stress Test, which includes both social and physical stress components. Choosing the amygdala as seed region, RSFC with the ventrolateral PFC, ventral PCC, cuneus, and culmen decreased after stress, whereas RSFC with the anterior hippocampal complex and the parahippocampal gyrus increased. Moreover, cortisol responders displayed stronger RSFC of the amygdala with the mPFC. During recovery, decreased RSFC was reported with the dorsolateral PFC (dlPFC), and ventral ACC, whereas an increase in RSFC was found for dACC and culmen. Again, differences between responders and non-responders were found: Responders were not only characterized by reduced connectivity with the left dlPFC, dACC, and culmen, but also by increased RSFC with the anterior hippocampal complex and the parahippocampal gyrus as compared with non-responders.

A more recent study applied the serial subtraction arithmetic task while comparing RSFC directly before and after stress induction (22). In contrast to the other studies, the authors refrained from a seed-based correlational analysis and instead, applied a data-driven approach. They reported strengthening of thalamo-cortical connectivity and weakening of cross-hemispheric parieto-temporal connectivity.

The last study focused on the late recovery phase from stress and studied amygdala RSFC 1 h after administration of the Trier Social Stress Task (23). Compared to a non-stressed control group, the stressed participants demonstrated increased amygdala RSFC with two cortical midline structures, the PCC/precuneus and mPFC. The authors concluded that the increased amygdala RSFC with the mPFC could represent top-down regulation of the amygdala by the mPFC, reflecting emotion regulation in the aftermath of stress. The increased amygdala RSFC with the PCC and precuneus was hypothesized to relate to memory consolidation of emotionally self-referential information, as those regions are involved in autobiographical memory processes (24, 25). An increase in RSFC between the amygdala and hippocampus was, contrary to the authors' expectations, not found in this study. In agreement with the study by Vaisvaser et al. (20), the amygdala RSFC pattern during late recovery from stress again points to effects of stress on the brain that stretch far beyond the immediate stress response.

In sum, previous research results showed divergent findings, which may be explained by differences in the type of stress induction, the experimental design, gender distribution of the study sample, and choice of seed regions. The focus of the current study was to examine the effects of psychosocial stress on amygdala RSFC in order to replicate and extend previous findings. For this purpose, moderate psychosocial stress was induced in healthy male and female volunteers inside the MRI scanner. To investigate the immediate recovery period from stress and its related functional connectivity patterns, RSFC of the amygdala was assessed during a resting-state fMRI scan acquired before and immediately after stress exposure. We expected that after stress the amygdala would demonstrate increased connectivity with regions involved in the down-regulation of the physiological stress response, in emotion regulation, and in memory consolidation. Moreover, as cortisol plays a key role in reaching homeostasis, we expected that these connections would be engaged differentially in people who demonstrate a cortisol increase in response to stress compared to those who do not.

## Materials and methods

### Participants

Hundred and four healthy volunteers were recruited through mailing lists of Berlin's universities, the experimental server platform PESA of the Humboldt-Universität zu Berlin, and through online advertorials. A compensation of 8 euro per hour was paid. An initial telephone-screening interview decided whether participants were eligible for inclusion or not. Exclusion criteria were: (history of) psychiatric diseases, as was checked by the screening questionnaire for Axis I disorders of the Structured Clinical Interview for DSM-IV (26), first-degree relatives with psychiatric diseases, contraindications for MRI scanning (e.g., metallic implants), acute or chronic neurological or physical diseases, history of alcoholism and/or drug abuse, current intake of prescription medication, color blindness, irregular sleep-wake rhythm, uncorrectable vision, and regular smoking (>5 cigarettes per day). Furthermore, students of psychology, medicine, or neuroscience were excluded because of potential previous knowledge about stress paradigms. Participants underwent a (neuro)psychological assessment containing the Edinburgh Handedness Inventory (27), Verbal Learning and Memory Test [VLMT; (28)], Multiple-Choice Vocabulary Intelligence Test [MWT-B; (29)], and German versions of the Beck Depression Inventory II [BDI-II; (30)], State-Trait Anxiety Inventory [STAI; (31)], Symptom Checklist-90 Revised [SCL-90-R; (32)], NEO-Five-Factor Inventory [NEO-FFI; (33)], Childhood-Trauma-Questionnaire [CTQ; (34)], as well as the English version of the Life-Events-Checklist [LEC; (35)]. The local ethics committee approved the study, and written informed consent was obtained from all participants.

Upon screening, a total of 104 participants were included in the study. Eighteen participants had to be excluded for the following reasons: Falling asleep during RS-fMRI (*n* = 7), severe image artifacts (*n* = 1), technical problems leading to delayed acquisition (*n* = 6), or early dropout from the study (*n* = 4). The final sample thus consisted of 86 participants (mean age 28.38 ± 7.25, range 20–58). Twenty-three females in our sample used contraceptives (19 oral contraceptive pill, 4 NuvaRing).

### Stress induction

To induce moderate psychosocial stress, a modified version of the Montreal Imaging Stress Task [MIST; (36)] was employed (ScanSTRESS) (37, 38). The stress paradigm is designed for use in a neuroimaging setting and combines social evaluative threat components (verbal and non-verbal feedback by the experimenters), as well as uncontrollable components (task difficulty, time constraints, and mock feedback of poor performance). Dickerson and Kemeny (39) demonstrated in a meta-analysis that stressors containing both these components lead to the strongest neuroendocrine stress response. Specifically, participants performed challenging arithmetic and mental rotation tasks under time pressure during stress blocks. Time for processing a trial was adapted to individual performance, thereby ensuring a low success rate in all participants. In addition, participants were continuously shown a live video stream of the two experimenters, who put on a critical and disapproving look to convey negative non-verbal feedback. After slow or incorrect responses, a text field indicating “Work faster!” or “Error!” appeared on the screen. During control blocks participants solved simple figure and number matching tasks without any time pressure, and without any visual or non-verbal feedback. The live video stream was crossed out and the two stressors did not observe the participants. For further details on the stress task, please refer to Dahm et al. (40).

### Physiological and subjective assessments of stress

**Figure 1:**
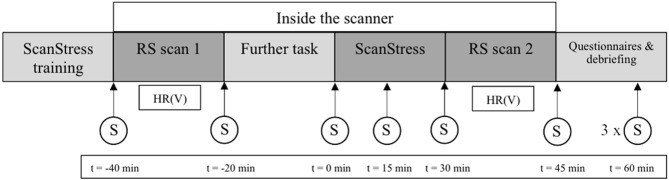
Procedure on the second day of scanning including the time-points of the salivary cortisol samples. Time (t) of sampling is relative to the onset of the ScanSTRESS task. The three last saliva samples were acquired every 15min; S, saliva sample; HR (V), heart rate and heart rate variability, RS, resting state.

To assess cortisol levels, nine saliva samples were collected throughout the procedure, using the Salivette saliva collection device (Sarstedt, Germany). In stress research, salivary cortisol is a common biomarker of psychological stress (41). Three saliva samples were collected before, one sample during, and five further samples after the stress task (see Figure 1 for an overview of the nine saliva sampling time-points). All samples were stored at −20°C until they were assayed at the Department of Biopsychology at the Technische Universität Dresden, Germany (https://tu-dresden.de/mn/psychologie/biopsychologie). To determine the cortisol concentrations in saliva (in nmol/l), the chemiluminescence-immuno-assay kit with high sensitivity (IBL, Hamburg, Germany) was employed. Inter- and intra-assay coefficients of variations were below 10%. For each participant, an aggregate measure of saliva cortisol secretion across all nine measurements was calculated: the area under the curve with respect to increase (in the following referred to as cortisol AUCi) (42). Positive values denote an increase in saliva cortisol over the course of the experiment, negative values a decrease.

**Figure 2:**
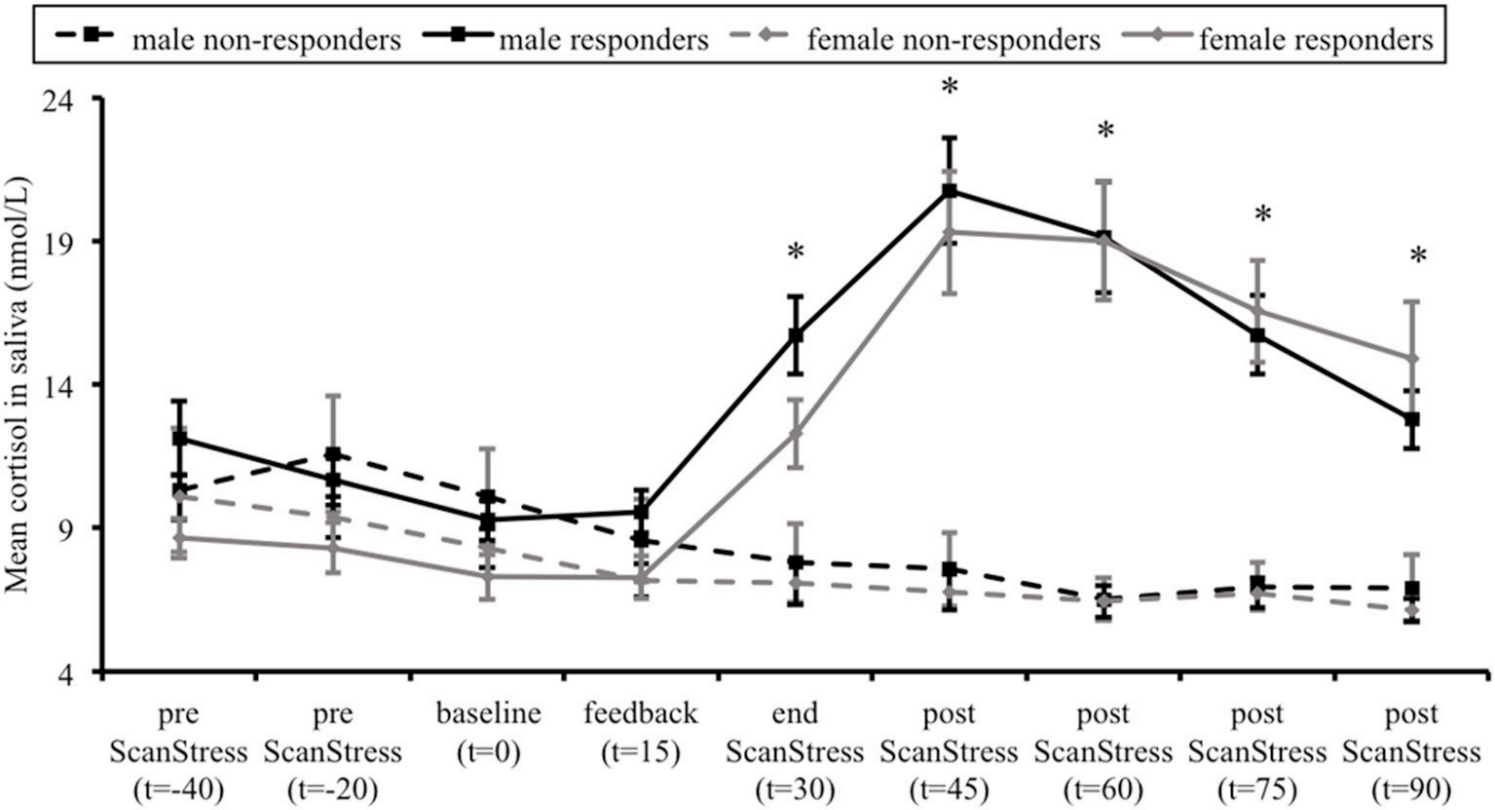
Mean salivary cortisol levels in cortisol responders and non-responders, for males and females separately. Error bars represent standard errors of the mean. Time (t) is relative to the onset of the ScanSTRESS task. ^*^p < 0.001, indicating difference between responders and non-responders across both sexes.

Before the statistical analysis of amygdala RSFC, participants were categorized as cortisol responder or non-responder based on a baseline-to-peak cortisol increase >2.5 nmol/l in response to the stress task (43). As saliva cortisol levels reach their peak 10–30 min after the end of stress induction (44), the difference between the sixth (15 min after the end of the stress task) and third saliva sample (immediately before the onset of the stress task) was calculated to characterize the stress-induced increase in cortisol. Averaged across all participants, these two sample time points also reflected the minimum before and the maximum absolute cortisol concentration after stress induction (see Figure 2). To test for existing baseline-differences, the groups were compared on all baseline physiological and psychometric measures. Because the assumption of normality was violated, non-parametric Mann–Whitney *U-*tests were used instead of unpaired *t*-tests.

Furthermore, heart rate was continuously recorded using an infrared pulse oximeter placed on the left ring finger (sampling rate of 50 Hz). The heart rate data acquired during the RS scans were used to determine parameters of heart rate frequency (HRF) and heart rate variability (HRV). For each RS scan, peak-to-peak intervals between the heartbeats were extracted using MATLAB R2012a (The Mathworks Inc.). Next, heart rate data were manually checked and corrected for misdetection and ectopic beats using the tool Physiological Noise Modeling (45). Finally, text files containing the corrected interbeat intervals of each RS scan were imported into KUBIOS HRV, a common software suite for HRV analysis (46). Besides HRF, this software calculates several distinct HRV parameters. The spectral power in the high-frequency band (0.15–0.4 Hz) during rest (HF-HRV) was used as a measure of HRV in further analysis, as it is known to reflect the vagal influence on cardiac function (47). That is, higher values indicate stronger parasympathetic activity. To achieve a normal distribution, the HF-HRV values were log-transformed.

Subjective stress experience during the ScanSTRESS task was assessed after the last saliva sample was acquired. Six items asked about negative affect during the task. Items were rated on a four-point scale ranging from “fully agree” to “fully disagree.”

HRF, HF-HRV, and cortisol were analyzed using three-way repeated measures ANCOVAs (mixed design) in SPSS Version 20 (IBM Corp.), with Group and Sex as between-subject factors, and Age as covariate, followed-up by relevant *Post hoc t*-tests. When the assumption of sphericity was violated, the degrees of freedom were corrected using the Greenhouse-Geisser adjustment. *Post-hoc t*-tests were conducted using Bonferroni adjusted alpha levels.

### FMRI data acquisition

Imaging data were acquired on a Siemens MAGNETOM TIM Trio 3.0 Tesla MRI scanner equipped with a 12-channel head coil (Siemens, Erlangen, Germany). For each RS scan, a total of 154 images was acquired using *T*${}_{2}^{*}$ weighted gradient-echo echo-planar imaging with the following scan parameters: 37 slices using an interleaved slice-acquisition in a descending order; repetition time (TR) = 2,020 ms; echo time (TE) = 25 ms; flip angle = 80°; field of view (FOV) = 192 × 192 mm; 64 × 64 matrix; 3 mm isotropic voxels with a 0.6 mm slice gap. Participants were instructed to lie still with their eyes closed in the darkened scanner room, not to think of anything in particular, and to stay awake during the entire scan.

For registration to standard space, a high-resolution anatomical image of the whole brain (voxel size 1 mm3) was obtained using a *T*_1_ weighted magnetization-prepared rapid gradient-echo (MP-RAGE) sequence with the following scan parameters: 192 sagittal slices; TR = 1,900 ms; TE = 2.52 ms; flip angle = 9°; FOV = 256 × 256 mm; 256 × 256 matrix; slice gap = 0.5 mm; parallel imaging technique GRAPPA acceleration factor 2. The scan took 4 min and 26 s. Each subject's anatomical image was inspected for abnormalities by a neuroradiologist.

### FMRI data preprocessing

The following preprocessing was carried out using FSL (48): motion correction, brain extraction, and spatial smoothing with a FWHM of 6 mm. Linear registration parameters were obtained for the functional-to-structural transformation, using FLIRT with the Boundary Based Registration (BBR) algorithm. Non-linear normalization parameters for the structural-to-standard-space (2 mm MNI) transformation were obtained with FNIRT, using the standard warp resolution setting of 10 mm. Next, functional data were further cleaned from artifacts using ICA-AROMA (49), which regresses out latent signal sources (independent components) that it classifies as noise. Lastly, a high-pass temporal filter of 125 s was applied to the cleaned 4D images, which were then normalized to standard space using the previously derived registration parameters.

### FMRI time-course extraction and statistical analysis

The goal of this study was to examine RSFC of the amygdala before and immediately after stress. For this purpose, a seed-based correlation analysis was employed. As a first step, binary masks of the left and right amygdala were anatomically defined by means of the Harvard Oxford Subcortical Probability Atlas, provided within FSL. Only voxels with ≥80% probability of belonging to the amygdala were used to create the seed masks. Next, these standard-space masks were registered to each participant's RS data set using the inverse of the MNI to native (fMRI) space transformation matrix. Afterwards, the first eigenvariate time series (i.e., most representative for all voxels within the mask) was extracted for each amygdala mask, for each participant, and for each RS scan separately. The same approach was applied to extract first eigenvariate time series of deep white matter (WM) and cerebrospinal fluid (CSF), to be used as covariates. Masks were created using FSL avg152 tissue priors (lower threshold value WM: 240, lower threshold value: CSF 160). For each seed and each RS scan, single subject general linear models (GLMs) were tested, including the seed's time-course as regressor of interest, together with the WM- and CSF-signal as covariate regressors. Four subject-level functional connectivity maps (left/right amygdala and pre/post stress) were thus obtained, representing voxels of which the time series were correlated with the time series of the seed. The functional connectivity maps were then fed into a second-level fixed effects analysis to calculate a difference (*z*-statistic) map between pre- and post-stress amygdala connectivity.

The difference maps between pre- and post-stress were assessed for each amygdala in an ANCOVA, with Group (responder and non-responder), Sex (males and females), and the interaction between those factors as between-subject variables, adding Age as covariate. The resulting *t*-statistical maps then underwent Threshold-Free Cluster Enhancement [TFCE; (50)], using the default parameter settings (*H* = 2, *E* = 0.5, *C* = 6), and significance testing was carried out with permutation testing (4,000 iterations) using the in-house developed *TFCE_mediation* software (51). In the latter step, a null distribution of random results was generated against which the empirical findings were tested, which resulted in statistical images that are family-wise error corrected for multiple comparisons at *p* < 0.05. Follow-up test between responders and non-responders were conducted for males and females separately, using the same settings mentioned above.

The test was repeated using a small volume correction for regions that were expected to change their connectivity with the amygdala in response to stress a priori. For this purpose, a mask containing the mPFC, the hippocampus, as well as the PCC and precuneus was created using the Harvard Oxford (Sub)Cortical Probability Atlas. Here we applied no probability threshold, to be as unbiased as possible. The same multiple comparison correction as before was then applied, but this time only for voxels falling inside the mask.

The ROI mask and (un)corrected statistical images are available on Neurovault (52) via this link: http://neurovault.org/collections/3578.

### General procedure

After study inclusion, participants completed three sessions on three separate days. At the first appointment, the absence of exclusion criteria was confirmed. Furthermore, the session contained a neuropsychological assessment, including tests for verbal memory and intelligence.

The last two sessions were conducted around and inside the MRI scanner. On the days of scanning participants were required to be awake for at least 4 h upon arrival, to refrain from caffeine and nicotine, not to eat 2 h before arrival, and to abstain from physical exercise after 7 pm on the day before. The MRI sessions took place on two consecutive days at the same time of day. Participants either arrived at 11:30 a.m. or 2 p.m. During the MRI sessions, participants had to complete three different task scans, and several anatomical scans were acquired as well. All scans relevant for the current study were acquired on the second day of scanning. On this day, participants first completed the training session of the ScanSTRESS task. Next, participants were brought into the scanner room, where the two RS scans, one before and one immediately after the stress task, were acquired. Throughout the procedure, nine saliva samples were collected. After scanning, participants completed several psychological questionnaires outside the scanner. After completing all questionnaires, participants were debriefed, thanked, and paid. The experimental procedure of the second day of scanning is shown in the Figure 1.

## Results

### Definition of cortisol responders and non-responders

Due to missing saliva samples, one female participant could not be assigned to either one of the two groups, and therefore was excluded. The classification resulted in 47 cortisol responders and 38 cortisol non-responders. Importantly, males were more likely to show a cortisol increase to the stress task than females [χ^2^ (1, *N* = 85) = 7.24, *p* = 0.007]. There was no significant difference between responders and non-responders for baseline cortisol concentrations, as well as for HRF and HRV before stress induction, independent of the factor Sex (all *p* > 0.05). Responders did show a higher mean score on the BDI-II, independent of the factor Sex. However, this difference between groups can be considered negligible, as the mean BDI scores of responders and non-responders were close to zero, far below the clinical cut-off (i.e., score > 13). Non-responders showed better performance during early recall on the VLMT than responders, though, on a descriptive level, between-group differences were small. Detailed between-group comparison results are reported in Tables 1, 2.

**Table 1:** Demographic, psychometric, and baseline cortisol characteristics of responders and non-responders.

	**Responders**		**Non-responders**		***z***	***p***
	***M***	***SD***	***M***	***SD***	
Gender (male/female)	26/21		10/28		
Handedness (right/left/both)	43/2/2		36/1/1		
School education (10 yr/ ≥ 12 yr)	3/44		2/36		
Higher education (no/university/other)	2/38/7		2/34/2		
Age	28.66	7.15	28.24	7.46	0.75	0.45
BDI-II	4.63	4.15	3.13	4.86	2.23	0.026
STAI-T	33.85	7.49	30.82	5.75	1.89	0.059
SCL-90-R Depression	1.91	3.59	1.52	3.06	1.19	0.23
SCL-90-R Anxiety	0.85	2.03	0.37	0.68	1.23	0.22
SCL-90-R Global Severity Index	0.22	0.18	0.17	0.16	1.05	0.29
NEO-FFI Neuroticism	15.07	6.01	13.05	6.13	1.28	0.20
LEC	5.17	3.68	5.16	4.01	0.21	0.84
CTQ	30.91	4.52	30.27	4.83	1.00	0.32
VLMT (hits early recall)	64.04	5.93	68.13	4.82	−3.39	0.001
VLMT (hits delayed recall)	13.72	1.73	13.79	2.43	−0.89	0.37
VLMT (recognition)	14.68	0.70	14.79	0.53	−0.68	0.50
MWT-B	31.36	2.37	31.50	2.44	−0.07	0.95
Baseline cortisol (nmol/L)	8.38	3.66	8.77	4.09	−0.29	0.77

*The Mann–Whitney U-Test was used for all group comparisons. BDI-II, Beck Depression Inventory II; STAI-T, State-Trait Anxiety Inventory-Trait; SCL-90-R, Symptom Checklist-90-Revised; NEO-FFI, NEO-Five-Factor Inventory; LEC, Life-Events-Checklist; CTQ, Childhood-Trauma-Questionnaire; VLMT, Verbal Learning and Memory Test; MWT-B, Multiple-Choice Vocabulary Intelligence Test*.

**Table 2:** Mean values and standard errors for HRF and HF-HRV before and after stress in responders and non-responders.

	**Responders**			**Non-responders**			***z***	***p***
	***M***	***SD***	***n***	***M***	***SD***	***n***	
**BASELINE**								
HRF	65.81	10.47	39	66.55	8.16	24	−0.28	0.777
HF-HRV^a^	6.83	1.04	39	6.78	0.97	24	0.28	0.777
**AFTER STRESS**								
HRF	70.37	12.58	41	68.13	9.72	26	0.55	0.58
HF-HRV^a^	6.44	1.12	41	6.57	1.26	26	−0.29	0.767

*The Mann–Whitney U-Test was used for all group comparisons. HRF, heart rate frequency; HF-HRV, heart rate variability, measured as spectral power in the high-frequency band (0.15–0.4 Hz) during rest; ^a^Values are log-transformed*.

### Results for physiological and psychological stress measures

Figure 2 illustrates the average saliva cortisol levels for each group at each sampling time-point. Over the course of the experiment, responders showed a substantial increase in cortisol levels, while non-responders exhibited a gradual decline. This was confirmed by a significant interaction between Group and Time, *F*_(2.58, 196.2)_ = 42.23, *p* < 0.001, $\eta_{partial}^{2}$ = 0.36. Further *Post hoc t*-tests did not reveal any group differences at baseline cortisol levels. As expected, responders' cortisol levels were higher at all time-points after the stress induction compared to non-responders (all *p* < 0.001). Importantly, there was neither a main effect of Sex on the cortisol levels, *F*_(1, 76)_ = 1.8, *p* = 0.18, $\eta_{partial}^{2}$ = 0.023, nor a Time × Sex, *F*_(2.58, 196.2)_ = 2.2, *p* = 0.099, $\eta_{partial}^{2}$ = 0.028, Group × Sex, *F*_(1, 76)_ = 0.017, *p* = 0.897, $\eta_{partial}^{2}$ < 0.001, or Group × Time × Sex interaction, *F*_(2.58, 196.2)_ = 0.696, *p* = 0.535, $\eta_{partial}^{2}$ = 0.009.

Besides differences in cortisol levels, responders (*M* = 11, *SD* = 4) reported significantly more negative affect during the stress task than non-responders (*M* = 8.57, *SD* = 4.01), *t*_(__82)_ = −2.76, *p* = 0.007, as did females (*M* = 10.73, *SD* = 3.79) compared to males (*M* = 8.86, *SD* = 4.45), *t*_(82)_ = −2.08, *p* = 0.041. Furthermore, higher subjective stress ratings were related to higher cortisol AUCi responses in responders irrespective of sex [*r*_(47)_ = 0.325, *p* = 0.026], and in females irrespective of being a responder or not [*r*_(48)_ = 0.523, *p* < 0.001].

Heart rate data were successfully acquired during 63 pre-stress and 67 post-stress RS scans. Table 2 provides the mean values of HRF and HRV before and after stress for responders and non-responders. Heart rate data for both RS scans were available for 57 participants (35 of them responders), which could thus be included in the further analysis of HRF and HRV. The repeated measures ANOVA for HRF showed a trend for the main effect of Time, *F*_(1, 52)_ = 3.91, *p* = 0.053, $\eta_{partial}^{2}$ = 0.07, indicating an increase in heart rate after stress in both groups. For the HF-HRV, a Group-by-Time interaction was revealed *F*_(1, 52)_ = 4.14, *p* = 0.047, $\eta_{partial}^{2}$ = 0.074. The group of responders drove this interaction, as they reacted with a decrease in HF-HRV in response to stress, *t*_(34)_ = 3.50, *p* = 0.001, while the HF-HRV of non-responders did not change. Furthermore, a main effect of Sex was found, *F*_(1, 52)_ = 8.95, *p* < 0.004, $\eta_{partial}^{2}$ = 0.15, indicating higher HF-HRV in women than in men. Lastly, stronger increases in HRF from pre- to post-stress were associated with higher cortisol in responders irrespective of sex [*r*_(35)_ = 0.376, *p* = 0.026], in females irrespective of being a responder or non-responder [*r*_(31)_ = 0.37, *p* = 0.04], and in male non-responders [*r*_(20)_ = 0.46, *p* = 0.041]. Stronger decreases in HF-HRV from pre- to post-stress were associated with higher cortisol in females irrespective of being a responder or non-responder [*r*_(31)_ = −0.465, *p* = 0.008].

Within females, there were not more non-responders taking contraceptives than responders, χ^2^ (1, *N* = 48) = 1.443, *p* < 0.23 (note that information was missing from one female). No differences were found between females with or without contraceptive medication regarding psychometrics or physiology, except that females without contraception were older (*p* = 0.008), had higher CTQ (*p* = 0.007) and MWT-B (*p* = 0.049) scores, and demonstrated a trend for higher AUCi (*p* = 0.067).

In summary, these findings indicate an effective stress induction, mostly in the group of responders, which was characterized by an increase in salivary cortisol levels, higher stress ratings, as well as an increase in HRF and a decrease in the HF-HRV.

### Resting-state functional connectivity results

**Figure 3:**
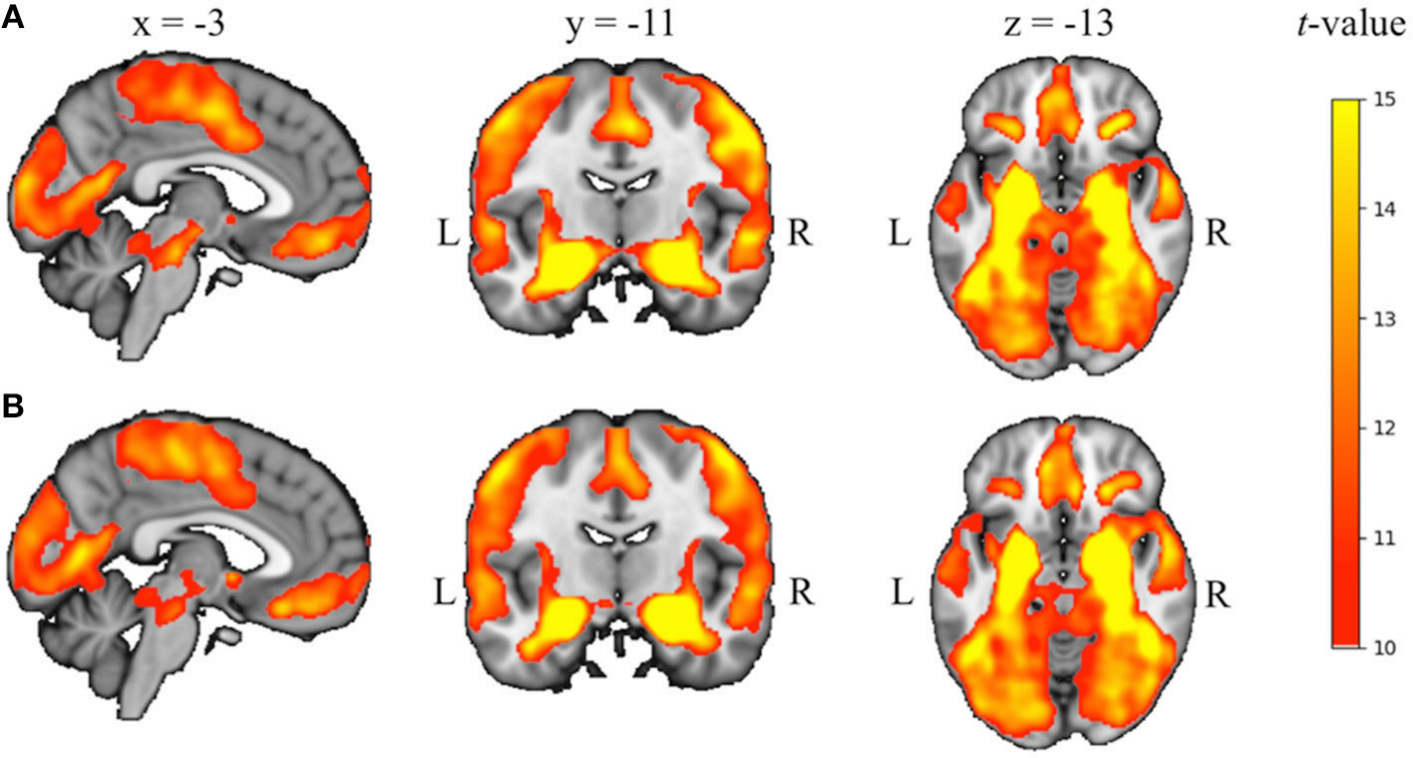
Seed-based correlation results across all participants and both RS scans for left **(A)** and right **(B)** amygdala, overlaid on the 2 mm isotropic 152-MNI standard space brain (*p* < 0.05, TFCE and FWE-corrected for multiple corrections). R, right, L, left.

For both hemispheres, the seed-based correlation analysis across all participants and both RS scans revealed a pattern of amygdala functional connectivity that was highly comparable to patterns previously reported in literature by ourselves and others (23, 53), including the medial prefrontal cortex, lateral orbitofrontal cortex, temporal poles, hippocampus, and brainstem (see Figure 3).

**Figure 4:**
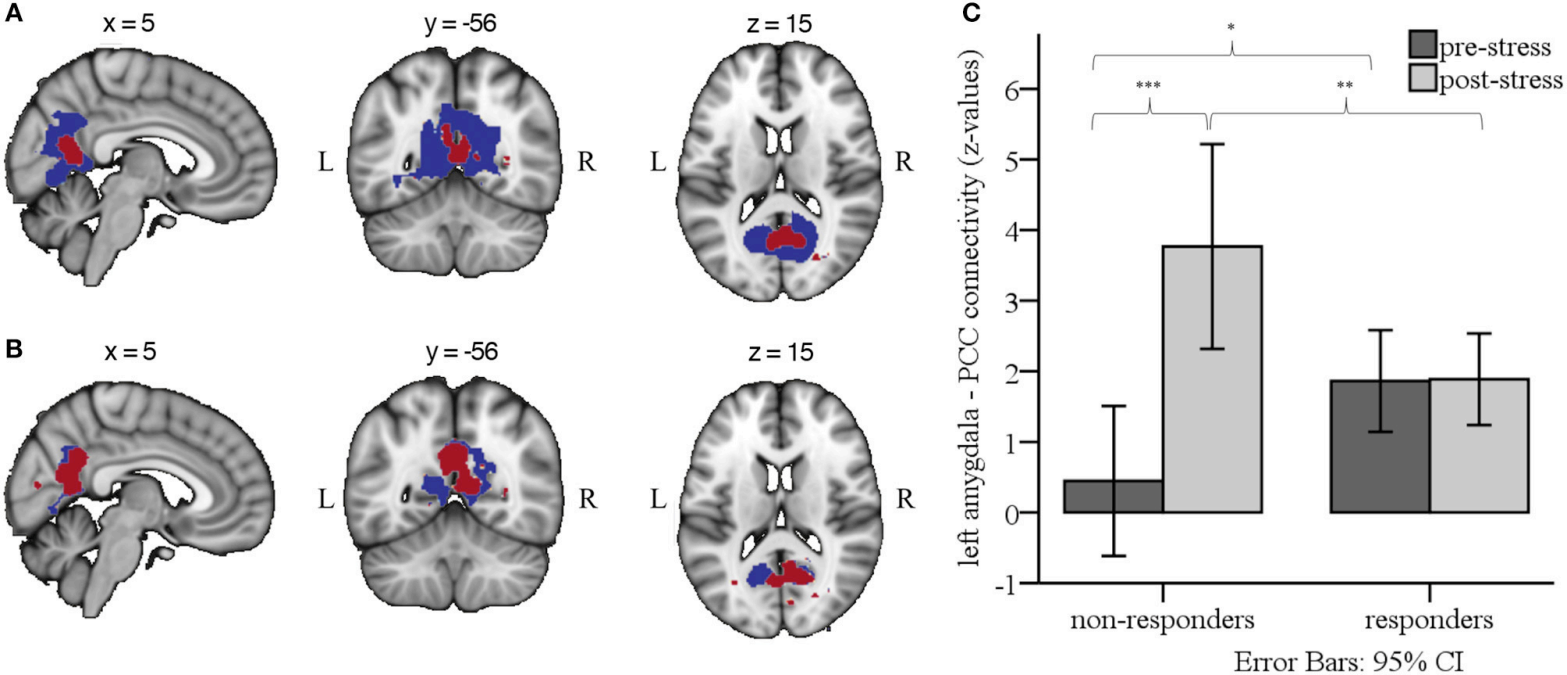
**(A)** Group-by-Sex-by-Time interaction effects for left (blue) and right (red) amygdala RSFC (*p* < 0.05, whole brain TFCE and FWE-corrected for multiple corrections). **(B)** Group-by-Time interaction effects for left (blue) and right (red) amygdala RSFC in males only, indicating enhanced RSFC from pre- to post-stress for non-responders compared to responders (*p* < 0.05, whole brain TFCE and FWE-corrected for multiple corrections). Results are overlaid on the 2 mm isotropic 152-MNI standard space brain. R, right; L, left. **(C)** Bar graph illustrating the Group × Time interaction effect for left amygdala RSFC, depicting mean *z*-values from each of the RS scans in male responders and non-responders. ^***^p < 0.001, ^**^p < 0.01, ^*^p < 0.05.

We found a significant Responder-by-Sex-by-Time interaction for both the left and right amygdala (see Figure 4A). Given the significant interaction and the unequal distribution of cortisol responders and non-responders between males and females, follow-up group level comparisons between responders and non-responders were carried out in males and females separately.

In male participants, we found a significant Group-by-Time interaction between the left and right amygdala seed and the PCC and precuneus (left amygdala connectivity peak: *x* = 10, *y* = −62, *z* = 20, cluster size = 2,129 voxels; right amygdala connectivity peak: *x* = 6, *y* = −62, *z* = 18, cluster size = 821 voxels; see Figure 4B). Specifically, *post hoc* comparisons on the individual extracted connectivity scores (*z*-values) between the two time-points confirmed an increase for RSFC between amygdala and PCC/precuneus in non-responders from pre- to post-stress, *t*_(9)_ = −6.95, *p* < 0.001, and stronger RSFC in non-responders than responders post-stress, *t*_(34)_ = 2.92, *p* = 0.006. Considering Bonferroni correction for four *post-hoc* tests, a trend was found for stronger RSFC pre-stress in responders than non-responders, *t*_(34)_ = −2.23, *p* = 0.033 (see Figure 4C).

The between-group analysis in female participants did not reveal RSFC of the left or right amygdala with any other brain region. Importantly, the results did not change when use of contraceptive medication was included as confound regressor in the statistical model.

To test whether interindividual differences in baseline cortisol might have confounded our results, we ran a follow-up analysis that included baseline cortisol concentrations (i.e., the third saliva sample) as covariate. The Responder-by-Sex-by-Time interaction, as well as the effects in males only, remained significant, and were even slightly more pronounced.

## Discussion

Using a seed-based correlation approach, we examined the effects of psychosocial stress on amygdala RSFC in healthy volunteers, as a function of the acute cortisol response. To this end, RS-fMRI data were acquired before and immediately after stress induction inside the MRI scanner. Participants were classified as either cortisol responder or non-responder. At baseline, there was no difference between the groups in salivary cortisol concentrations. We found no differences between male and female participants in respect to cortisol levels. However, males were more likely to show a cortisol increase to the stress task than females. Furthermore, responders reported higher negative affect during the stress task than non-responders, while negative affect was associated with cortisol AUCi. Responders also demonstrated a decrease in HRV in the high-frequency range (HF-HRV) in response to stress, whereas non-responders showed no change. A decline of the HF-HRV points to a decline in vagal activity, and an increase in sympathetic influence (47). Thus, the decline of HF-HRV found in responders likely reflects a sustained autonomic arousal in the group of responders following the stress task. Taken together, the physiological and behavioral measures confirmed successful stress induction in the cortisol responders as compared to non-responders.

Based on the group differences in physiological stress reactivity, we expected amygdala RSFC to be differentially affected in responders and non-responders following stress as well. As responders and non-responders were distributed differently in males and females, with males being more likely to show a cortisol increase to the stress task than females, and the full factorial analysis demonstrated a significant Responder-by-Sex-by-Time interaction, group level comparisons between responders and non-responders were carried out in males and females separately. Whereas, we did not detect changes in amygdala RSFC between female responders and non-responders, the results showed a significant Group-by-Time interaction in males, demonstrating an increase in bilateral amygdala RSFC with the PCC and the adjacent precuneus from pre- to post-stress in non-responders, but not in responders. So far, previous studies that distinguished between responders and non-responders reported differential RSFC between other brain regions. Vaisvaser et al. (20), for example, found increased connectivity between the hippocampus and amygdala up to 2 h for non-responders. Quaedflieg et al. (21) reported increased FC between amygdala and dlPFC, dACC, and culmen, but decreased FC with the anterior hippocampal complex and the parahippocampal gyrus.

Considering that our connectivity analysis across participants and RS scans demonstrated strong RSFC between the amygdala and hippocampus, the RSFC between the amygdala and PCC/precuneus found here could in fact be driven or mediated by the hippocampus, a key region for storage and retrieval of episodic information (54). As the amygdala and hippocampus are bordering each other, and as the fMRI resolution is not high enough to completely disentangle signal from the amygdala and the anterior part of the hippocampus, the amygdala signal could be contaminated by signal from this area. Further, given that the hippocampus and the precuneus are directly connected through white matter pathways (55), amygdala RSFC with these regions may, in part, reflect connectivity of the hippocampus. This is, however, still in line with the assumption that increased RSFC with the PCC/precuneus after stress might be related to memory consolidation of emotionally salient information. The possibility that amygdala RSFC may have been mixed with hippocampal RSFC could also explain why we did not find any interaction effect for amygdala RSFC with the hippocampus. That is, artificially high functional connectivity may have been induced between the amygdala and hippocampus due to autocorrelation. This, in turn, could have obscured any underlying differences in connectivity between the two groups.

Consistent with the findings of the previous study of Veer et al. (23), the amygdala was coupled with the PCC/precuneus, a core component of the default mode network (DMN) (56). It should be noted, though, that the previous and current study diverge from each other not only in the time point of the assessment after stress, but also in the specific group that showed an effect. Whereas, the previous study assessed RSFC an hour after stress and reported increased FC in a stressed group compared to a non-stressed group, irrespective of being responder or non-responder, we assessed RSFC immediately after stress in the current study and found increased FC in non-responders compared to responders, as all participants were exposed to the stress task. The lack of a non-stressed control group and a second assessment at a later stage of recovery in the current study hampers the comparison of results, though we could hypothesize that responders might have shown a similar increase of RSFC, but at a later time point. This would suggest that responders and non-responders might exhibit a different time line in terms of their neural response after psychosocial stress, which should be considered in future research.

The DMN is known to be implicated in several functions related to the self, including mind wandering (57), self-referential thought (58–60), autobiographical memory, as well as integrating past, present, and future experiences (61). Considering that DMN regions are functionally and structurally interconnected (55), the connectivity pattern between the amygdala and the PCC/precuneus could indicate an increased engagement of the amygdala within the DMN directly after stress in non-responders. Several explanations could underlie this finding: First, the stress paradigm we used may have been unable to induce stress in our non-responding participants. This could, for example, stem from earlier experiences with similar stressful situations, which then have led to a higher threshold of stress reactivity for this particular class of stressors (62). Accordingly, as Veer et al. (23) reported RSFC between amygdala and PCC/precuneus across all stressed participants an hour after stress, independent of cortisol responsivity, our results could mean that non-responders are able to activate this specific circuit more rapidly than responders, which could facilitate immediate updating of memory schemata by integrating recent experiences. Conversely, not responding to the stress task with an increase in cortisol might relate to maladaptive stress processing. For example, previous studies showed that a blunted cortisol response emerges in people who experienced adverse life-events (63) or, although preliminary, in schizophrenia patients (2). However, irrespective of whether a lack of a cortisol response should be considered maladaptive, reactions to stressful situations depend on many different factors, as Bonanno and Burton (64) discussed with their concept of “regulatory flexibility.” Coping with stressors and regulating one's emotions properly is a dynamic process, which gives every individual a chance to adapt to adverse events in his or her own range of capabilities in the time they need.

It is important to note that the findings of the current study do not necessarily diverge with the results obtained in related studies in healthy volunteers that examined the effects of stress on brain functional connectivity. As van Marle et al. (19) used a different kind of stressor, containing neither social evaluative threat nor uncontrollable components, it is plausible that their stressor triggered a qualitatively different stress response, both physiologically and psychologically, and therefore different recovery processes compared to the stressor in our study. Although the study design of Vaisvaser et al. (20) was quite similar to the design of the current study, the selection of different seed regions makes it hard to compare our results to theirs. However, convergent with the current findings, Vaisvaser et al. did find sustained effects of stress on RSFC in non-responders. In contrast, Quaedflieg et al. (21) did use a similar design and the amygdala as seed region, yet conducted their analysis across female and male participants. They reported increased RSFC with dorsolateral PFC, dorsal ACC, and culmen in non-responders after stress. This illustrates that stress has prolonged effects on brain function, which might be related to adaptive recovery from a stressful situation.

### Limitations and future directions

There are several limitations. First, data were acquired within a larger fMRI study, in which a fear extinction paradigm [adapted from Phelps and colleagues; (65)] was administered after the first resting-state scan, but prior to the ScanSTRESS task. We thus cannot rule out that the fear extinction paradigm had some effect on the stress reactivity of our participants. However, this task did not contain any aversive stimulation, as was used during fear acquisition on the day before. Further, all participants showed successful extinction learning, both directly after conditioning on day 1 of the study, as well as during late extinction prior to the stress task, as was confirmed by attenuation of the skin conductance response. Thus, it is quite likely that this task had rather negligible effects on stress reactivity.

Second, the study design did not include a non-stress control group. All participants underwent the stress task, and were classified *post hoc* as either cortisol responder or non-responder. This approach has the disadvantage that we could only reveal differences in amygdala RSFC related to cortisol differences between the groups, and not necessarily related to experiencing stress *per se*. In this context, it would be interesting to assess whether there are measurable differences in RSFC between cortisol non-responders and non-stressed controls that could account for non-hormonal effects of stress on amygdala RSFC.

Third, the difference in stress response between women and men found in the current study are in line with previous reports. In general, men tend to show larger salivary cortisol increases in response to a psychological stress task than women (66). Studies suggest that age (67), the use of contraceptives and phase of menstrual cycle (68–70), as well as sex hormones (71) contribute to differences in cortisol response. In our sample, 23 female participants took contraceptives. However, this could not account for the differences in any of our dependent variables. The number of days between the onset of the last menstrual cycle until the MRI assessment was enquired, but not individual cycle durations. As such, we could not estimate the exact menstrual phase of our female participants and test for its effects in our connectivity analyses. Furthermore, the ScanSTRESS task much relies on uncontrollable failure (i.e., achievement stress), which seems to affect men especially (72). This might thus have caused female participants to demonstrate a smaller stress response than males. Lastly, the composition of the stress panelists in terms of their sex was found to have an influence on the neuroendocrine stress response in both men and women (73). The authors reported cortisol increases only if the panel consisted of opposite sex members. Although we used male and female panelists, the panel in our study neither was composed of women and men in a consistent manner, nor was it composed depending on the subjects' sex. Additionally, we had more female than male panelists, which could explain the higher responder rate among the male subjects.

Fourth, it should be noted that our results are limited to amygdala-based circuits only, given the seed-based approach used. Surely, stress affects many other brain regions, so there is a fair chance that we have missed changes in functional connectivity that emerged independent of the amygdala. Nonetheless, as the amygdala plays a pivotal role in most central stress-related processes, the selection of the amygdala as a seed is reasonable, and has provided a good insight in the role of stress-related brain circuits during recovery from stress.

Last, further studies are warranted to replicate the findings of the current study, and compare these to a control group. For this, one challenge would be to find an equivalent task for the control group, which does not induce any stress, but still is comparable to the stress task.

## Conclusion

Taken together, the results of the current study add to the growing body of literature addressing the immediate recovery from stress. The neural circuits involved contain brain regions that are implicated in the regulation of the physiological stress response, in emotion regulation, and in memory consolidation, which underscores the necessity of these processes in recovering from stress. The current study extends findings from previous studies, which demonstrated differences in RSFC between cortisol responders and non-responders as well (20, 21). Together with studies that compared effects between a stressed and control group, these findings can provide a preliminary time line spanning both the immediate and long-term recovery from psychosocial stress. Interestingly, the results suggest a mediating role of cortisol on amygdala-posterior midline connectivity in the aftermath of stress. Although this study was carried out in healthy participants, and the results likely reflect normal variations in the neural response to stress, understanding the mechanisms that underlie these variations could prove beneficial in revealing neural markers that promote resilience to stress-related disorders.

## Ethics statement

This study was carried out in accordance with the recommendations of and after approval from the Medical Ethics Committee of Charité-Universitätsmedizin Berlin. All participants gave written informed consent in accordance with the Declaration of Helsinki.

## Author contributions

SE, HW, PF, and IV were involved in the development of the study. PF, KD, and IV were involved in the data collection. AD, IV, KD, and SE were involved in the analysis and interpretation of data. All authors gave final approval of the version to be published, and agreed to be accountable for all aspects of the work.

### Conflict of interest statement

The authors declare that the research was conducted in the absence of any commercial or financial relationships that could be construed as a potential conflict of interest.
